# Role of the chemokine receptor CCR5-dependent host defense system in *Neospora caninum* infections

**DOI:** 10.1186/s13071-014-0620-5

**Published:** 2015-01-06

**Authors:** Chisa Abe, Sachi Tanaka, Maki Nishimura, Fumiaki Ihara, Xuenan Xuan, Yoshifumi Nishikawa

**Affiliations:** National Research Center for Protozoan Diseases, Obihiro University of Agriculture and Veterinary Medicine, Inada-cho, Obihiro, Hokkaido 080-8555 Japan; Faculty of Agriculture, Shinshu University, Minami-Minowa, Kamiina Nagano, 399-4598 Japan

**Keywords:** Chemokines, Dendritic cells, Neuroimmunology, Parasitic-Protozoan

## Abstract

**Background:**

*Neospora caninum*, a *Toxoplasma gondii*-like obligate intracellular parasite, causes abortion in cattle and neurological signs in canines. To understand neosporosis better, studies on host cell migration and host immune responses during the early phase of infection are important. Although the C-C chemokine receptor 5 (CCR5) plays a crucial role in immune cell migration, the role played by it in protective immunity against *N. caninum* is poorly understood.

**Methods:**

CCR5^−/−^ mice were used to investigate their sensitivity levels to *N. caninum* infection and their ability to activate immune cells against this parasite.

**Results:**

Increased mortality and neurological impairment were observed in the *N. caninum*-infected CCR5^−/−^ mice. In comparison with wild-type mice, CCR5^−/−^ mice experienced poor migration of dendritic cells and natural killer T cells to the site of infection. Dendritic cells in an *in vitro* culture from CCR5^−/−^ mice could not be activated upon infection with *N. caninum*. Furthermore, higher levels of IFN-γ and CCL5 expression, which are associated with brain tissue damage, were observed in the brain tissue of CCR5^−/−^ mice during the acute phase of the infection, while there was no significant difference in the parasite load between the wild-type and CCR5^−/−^ animals. Additionally, a primary microglia culture from CCR5^−/−^ mice showed lower levels of IL-6 and IL-12 production against *N. caninum* parasites.

**Conclusions:**

Our findings show that migration and activation of immune cells via CCR5 is required for controlling *N. caninum* parasites during the early phase of the infection.

**Electronic supplementary material:**

The online version of this article (doi:10.1186/s13071-014-0620-5) contains supplementary material, which is available to authorized users.

## Background

*Neospora caninum* is an obligate intracellular apicomplexan parasite. This parasite is a cause of neosporosis, which leads to abortion, neonatal mortality and congenital infection in cattle, and neuromuscular signs in canines [1]. Calves infected vertically also have neurologic signs including hind limbs that are flexed and hyperextended, and loss of conscious proprioception [2]. The ability of a host to survive an infection with *N. caninum* is IFN-γ-dependent [3]. IFN-γ is a known major mediator of resistance against *Toxoplasma gondii*, a parasite closely related to *N. caninum* [4]. IL-12 stimulates production of IFN-γ from natural killer (NK) cells, CD4^+^ cells and CD8^+^ cells [5]. In one study, *N. caninum* infection induced IL-12 synthesis by dendritic cells and macrophages, suggesting that IFN-γ-secretion by T lymphocytes in combination with IL-12 production occurred following interactions between T cells and antigen-presenting cells [6].

Chemokines are a large family of chemotactic proteins, which regulate leukocyte activation and recruitment to sites of inflammation via interaction with a family of chemokine receptors [7]. Cystein–cystein chemokine receptor 5 (CCR5) and its ligands, such as macrophage inflammatory protein-1 alpha (MIP-1α) and beta (MIP-1β), play a role in IFN-γ generation during the early phase of infection with *Leishmania donovani* [8]. CCR5-deficiency in mice decreases susceptibility to experimental cerebral malaria infection [9], suggesting that interactions between host CCR5 and malaria parasites are important for parasite control of the infection. During *T. gondii* infection, *T. gondii* cyclophilin 18 (TgCyp18) was found to induce IL-12 production through binding to CCR5 in a CCR5-dependent manner [10,11]. In the case of *N. caninum*, excreted and secreted antigens triggered monocytic cell migration to the site of infection in a CCR5-dependent manner [12]. Moreover, *N. caninum* cyclophilin caused CCR5-dependent migration of murine and bovine cells [13]. Thus, CCR5 regulates the type of immune cell migration and cytokine production required for host control of parasites in *T. gondii* and *N. caninum* infections. However, the role played by CCR5 in protective immunity against *N. caninum* has not been clarified as yet. In this study, we investigated the sensitivity levels and degree of neurological impairment of CCR5^−/−^ mice infected (intraperitoneally) with *N. caninum* to obtain better understanding of the role of CCR5-dependent host immunity.

## Methods

### Ethics statement

This study was performed in strict accordance with the recommendations in the Guide for the Care and Use of Laboratory Animals of the Ministry of Education, Culture, Sports, Science and Technology, Japan. The protocol was approved by the Committee on the Ethics of Animal Experiments of the Obihiro University of Agriculture and Veterinary Medicine (Permit number 25–59, 24–15, 23–61). All surgery for sampling of cardiac puncture blood, tissues, bones and ascites was performed under isoflurane anesthesia, and all efforts were made to minimize animal suffering.

### Mice

C57BL/6 J mice, 5–8 weeks of age, were obtained from Clea Japan (Tokyo, Japan). CCR5 knockout (CCR5^−/−^) mice (B6.129P2-Ccr5^tmlKuz^/J, Stock No. 005427) were purchased from the Jackson Laboratory (Bar Harbor, Maine, USA). The mice were housed under specific pathogen-free conditions in the animal facility of the National Research Center for Protozoan Diseases at the Obihiro University of Agriculture and Veterinary Medicine, Obihiro, Japan.

### Parasites and *in vivo* infections

*N. caninum* (Nc-1 isolate) tachyzoites and its recombinants expressing the GFP were maintained in monkey kidney adherent epithelial cells (Vero cells) cultured in Eagle’s minimum essential medium (EMEM, Sigma, St Louis, USA) supplemented with 8% heat-inactivated fetal bovine serum (FBS). To purify tachyzoites, parasites and host cell debris were washed with PBS, after which the final pellet was resuspended in PBS and passed through a 27-gause needle and a 5.0-μm-pore-size filter (Millipore, Bedford, MA, USA). Male mice were experimentally infected by the i.p. route with 1 × 10^6^ tachyzoites per mouse. All mice were monitored for survival and scored on a daily basis for the neurological signs characteristic of neosporosis, including torticollis and circling motion. Clinical score-assessed neurological signs such as torticollis and circling motion scored 1 point each. Dead mice showing neurological signs were assigned a maximal score of 2. The scores were assessed using a modified set of criteria adapted by Reichel and Ellis [14].

### Quantitation of parasite burden

For DNA preparation, brain, lung, liver, and spleen were collected, frozen at −80°C, and resuspended in ten weight equivalent volumes of extraction buffer (0.1 M Tris–HCl pH 9.0, 1% SDS, 0.1 M NaCl, and 1 mM EDTA) and 100 μg/ml of Proteinase K at 50°C. DNA was purified by phenol–chloroform extraction and ethanol precipitation. For each tissue, the DNA concentration was adjusted to 50 ng per μl and 1 μl was used as template DNA. Parasite DNA was quantified as described previously [15]. Oligonucleotide primers were designed to amplify a 76-bp DNA fragment of the *N. caninum* Nc5 sequence (GenBank accession no. X84238). The *N. caninum* Nc5 forward primer spans nucleotides 248 to 257 (5’-ACT GGA GGC ACG CTG AAC AC-3’) and the *N. caninum* Nc 5 reverse primer spans nucleotides 303 to 323 (5’-AAC AAT GCT TCG CAA GAG GAA-3’). PCRs (25-μl total volume) contained 1 × SYBR Green PCR Buffer, 2 mM MgCl_2_, a 200 μM concentration each of dATP, dCTP, and dGTP, 400 μM dUTP, 0.625 U of AmpliTaq Gold DNA polymerase, and 0.25 U of AmpErase UNG (urasil-N-glycosilase) (all of which are included in the Power SYBR Green PCR Master Mix, PE Applied Biosystems, Foster City, CA, USA); additionally, 20 pmol of each primer (Amersham Pharmacia Biotech, Inc., Piscataway, NJ) and 1 μl of template DNA were added. Amplification was performed by a standard protocol recommended by the manufacturer (2 min at 50°C, 10 min at 95°C, 40 cycles at 95°C for 15 s, and 60°C for 1 min). Amplification, data acquisition, and data analysis were performed by the ABI 7700 Prism Sequence Detector (Applied Biosystems, Foster City, CA, USA), and the cycle threshold (Ct) values calculated were exported to Microsoft Excel for analysis. A standard curve was established from *N. caninum* DNA extracted from 1 × 10^5^ parasites using 1 μl samples of serial dilutions ranging from 10,000 to 0.01 parasites. Parasite numbers were calculated by interpolation of the standard curve, with the Ct values plotted against a known concentration of parasites. To confirm the specificity of the PCRs, DNA from the brain of an uninfected mouse and from purified *N. caninum* tachyzoites were used as the negative and positive controls, respectively. The limit of detection was 0.1 parasites in 50 ng of tissue DNA.

### Real-time RT-PCR analysis

Total RNA was prepared from brain and liver samples from the CCR5^−/−^ (*N* = 10) and C57BL/6 mice (*N* = 10) using TriReagent™ (Sigma, USA) according to the manufacturer’s instructions. First-strand cDNA synthesis used an oligo (dt) primer and RT-superscript II (Invitrogen, Carlsbad, CA, USA) reverse transcriptase. PCR was performed as described above, using Power SYBR Green PCR Master Mix and an ABI 7700 Prism Sequence Detector instrument. The relative mRNA amounts were calculated using the comparative C_T_ method (User Bulletin no. 2, Perkin-Elmer). The primer sequences (sense and antisense sequences) designed by Primer Express Software (Applied Biosystems, Foster City, CA, USA) were as follows: β-actin sense primer 5’-GCT CTG GCT CCT AGC ACC AT-3’, β-actin antisense primer 5’-GCC ACC GAT CCA CAC AGA GT-3’, glyceraldehyde-3-phosphate dehydrogenase (GAPDH) sense primer 5’-TGT GTC CGT CGT GGA TCT GA -3’, GAPDH antisense primer 5’- CCT GCT TCA CCA CCT TGT TGA T-3’, mouse IFN-γ sense primer 5’-GCC ATC AGC AAC AAC ATA AGC GTC-3’, mouse IFN-γ antisense primer 5’-CCA CTC GGA TGA GCT CAT TGA ATG-3’, human CCL5 sense primer 5’-GCT TGC AAA CAC CTG ATG TCC-3’, human CCL5 antisense primer 5’-CCC TTC TCG GAG AGC TTT TGT-3’, TNF-α sense primer 5’-GGC AGG TCT ACT TTG GAG TCA TTG C-3’, TNF-α antisense primer 5’-ACA TTC GAG GCT CCA GTG AA-3’. Gene-specific expression was normalized against β-actin and GAPDH housekeeping gene expression. The optimal reference gene was selected based on the Cotton EST database (http://www.leonxie.com).

### Pathological analysis

After fixation, the coronally cut liver, spleen, lung and brain tissue samples were embedded in paraffin wax, sectioned to 4 μm, and then stained with hematoxylin and eosin. To estimate the severity of the histopathological lesions in the brain, they were scored using the following scheme: 0, no lesion; 1, minimal lesions limited to localized perivascular cuffs or slight mononuclear cell infiltration in the meninges; 2, mild lesions, including perivascular cuffs, meningitis and local glial cell infiltration; 3, moderate lesions, including perivascular cuff, meningitis, glial cell infiltration, focal necrosis and rarefaction of the neuropil with occasional macrophage infiltration; 4, severe lesions, including perivascular cuffs, meningitis, glial cell infiltration, rarefaction of the neuropil and extensive necrosis. The scores for each lesion were added for each section, and the total pathological score for each section was used in the data analysis. We scored one section that including some pieces of brain tissue cut coronally in each mouse.

### Preparation of peritoneal cells

Peritoneal exudate cells from the mice were harvested by lavage with 5 ml of ice-cold PBS. The cells were filtered through a 40-μm cell strainer to remove cell aggregates and small pieces of debris. The cells were centrifuged at 1,000 × *g* for 5 min, suspended in PBS and used for flow cytometric analysis.

### Flow cytometric analysis and antibodies

Cells were prepared for fluorescence activated cell sorting analysis as described below. Following removal of the culture medium, the cells were washed with PBS and resuspended in cold PBS containing 0.5% bovine serum albumin. The cells were treated with FcBlock™ to avoid the non-specific adherence of mAbs to Fc receptors, and then incubated with their respective monoclonal antibodies (Additional file 1: Table S1) for 15 min at 4°C. The stained cells (monocyte/macrophage; CD11b^+^ CD11c^−^, dendritic cell; CD11b^−^ CD11c^+^, neutrophil; Gr-1^+^ MHC class II^−^, natural killer (NK) cell; CD3^−^ NK1.1^+^, NKT cells; CD3^+^ NK1.1^+^, T cell; CD3^+^) were washed with cold PBS, fixed with 0.5% paraformaldehyde in PBS, and examined with an EPICS XL flow cytometer (Beckman Coulter, Hialeah, USA). *N. caninum*-infected cells were GFP^+^ by flow cytometry. The absolute number of each cell marker was calculated as follows: the absolute cell number = the total host cell number × (the percentage of marker^+^ cells/100) × (the percentage of gated cell by the flow cytometry/100).

### Preparation of peritoneal macrophages for *in vitro* studies

CCR5^−/−^ and C57BL/6 mice were injected i.p. with 1 ml of 4.05% thioglycolate. Four days after these injections, peritoneal exudate cells were harvested from the mice by lavage with 5 ml of ice-cold PBS and depleted of red blood cells with 0.83% NH_4_Cl and 0.01 M Tri-HCl, pH 7.2. Cells were centrifuged at 1,000 × g for 10 min and suspended in DMEM (Sigma) supplemented with 10% FBS. The macrophage suspension was then added to a 12-well plate at 1 × 10^6^ cells/well. After 24 h incubation, the macrophages (1 × 10^6^ cells) were infected with 2 × 10^5^  *N. caninum* tachyzoites and incubated for 24 h for *in vitro* analysis. Cells were found to be 97% macrophages, as judged by positive staining for CD11b.

### Preparation of bone marrow-derived dendritic cells (BMDCs)

BMDCs were prepared by a reported method [16] with some modifications. After removing all muscle tissues from the mouse femurs and tibias, the bones were placed into a fresh dish with RPMI 1640 medium (Sigma). Both ends of each bone were cut with scissors and the bone marrow cells were flushed out with a syringe and a 25-gause needle using RPMI 1640 medium. Following lysis and red blood cell removal, the cells were resuspended in RPMI 1640 supplemented with 10% FBS and 10 ng/ml of murine recombinant granulocyte-macrophage colony-stimulating factor (GM-CSF) (R&D Systems, Minneapolis, MN, USA) and cultured in a 24-well plate at 5 × 10^5^ cells/well at 37°C. The cells were cultured for 7 days and the supernatants were gently removed and replaced with fresh media every 48 h. On day 8 of culturing, mature and loosely attached BMDCs (5 × 10^5^ cells) were infected with 2 × 10^5^ tachyzoites and incubated for 24 h for *in vitro* analysis.

### Preparation of microglia cultures

Murine microglia were cultured from the brain cortices of neonatal mice (age, E17-18), following the procedure previously described [17], with some modifications. Pups were decapitated and their brains were removed, the cortices were dissected, and the meninges were removed. Tissues were mechanically dissociated into a single-cell suspension in DMEM containing 0.25% trypsin and 0.01% DNase at 37°C for 10 min. After washing, the cells were resuspended in DMEM/F-12 (Gibco, Carlsbad, CA, USA) supplemented with penicillin–streptomycin (0.5 mg/ml), 10% FBS, and 10 ng/ml of GM-CSF, and then plated into 75-cm^2^ flasks at 4 × 10^6^ cells. The cultures were incubated at 37°C. Cell culture medium was changed thereafter every three days. After 10 to 11 days, microglial cells were detached from the astrocyte monolayer by pipetting. The supernatants were collected and centrifuged, and the cells were reseeded on a 24-well plate at 2 × 10^5^ cells/well. Microglial cells were allowed to grow for an additional 16 h before the experiments were started. The microglial cells (2 × 10^5^ cells) were infected with 2 × 10^5^ tachyzoites and incubated for 24 h for *in vitro* analysis. Cells were found to be 95% microglia as judged by positive staining for CD11b.

### Cytokine enzyme-linked immunosorbent assay (ELISA)

IL-6 and IL-12 p40 levels in the culture supernatant of peritoneal macrophages, BMDCs and microglia and in the sera and ascites of mice were measured by an OptEIA™ Mouse IL-6 or IL-12 (p40) ELISA Set (BD Bioscience, San Jose, CA, USA), respectively, according to the manufacturer’s instructions.

### Statistical analysis

The various assay conditions used herein were evaluated with a Student’s *t*-test or ANOVA test followed by Tukey’s multiple comparisons procedure. The statistical significance of differences in mouse survival was analyzed with a Kaplan–Meier nonparametric model and the curves were compared using the log-rank test.

## Results

### Survival rates and clinical scores of CCR5-deficient mice infected with *N. caninum*

**Figure 1:**
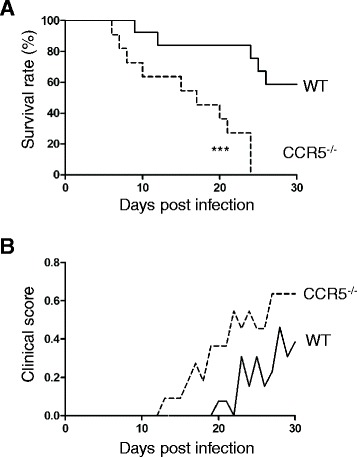
**Survival rate and clinical score of mice following lethal challenge with** ***N. caninum*** **. (A)** Survival rates of *N. caninum*-infected CCR5^−/−^ and C57BL/6 mice (wild-type, WT). Data were analyzed by a log-rank test. *** *P* < 0.001. **(B)** Clinical scores represent the mean total values for all mice used in this study. Data were obtained from two independent experiments performed together (CCR5^−/−^ mice, *N* = 5 + 6; C57BL/6 mice, *N* = 6 + 7).

CCR5^−/−^ mice showed significantly higher mortality rates than C57BL/6 mice (Figure 1A). More than 60% of the C57BL/6 mice survived whereas all CCR5^−/−^ mice succumbed to the infection. CCR5^−/−^ mice also showed higher weight loss compared with C57BL/6 mice after the infection. Moreover, higher clinical scores assessing the severity of the neurological signs (e.g., torticollis and circling motion), which occurred at an earlier stage of the infection, were observed in the CCR5^−/−^ mice than in the C57BL/6 mice (Figure 1B).

### Parasite tissue burden

**Figure 2:**
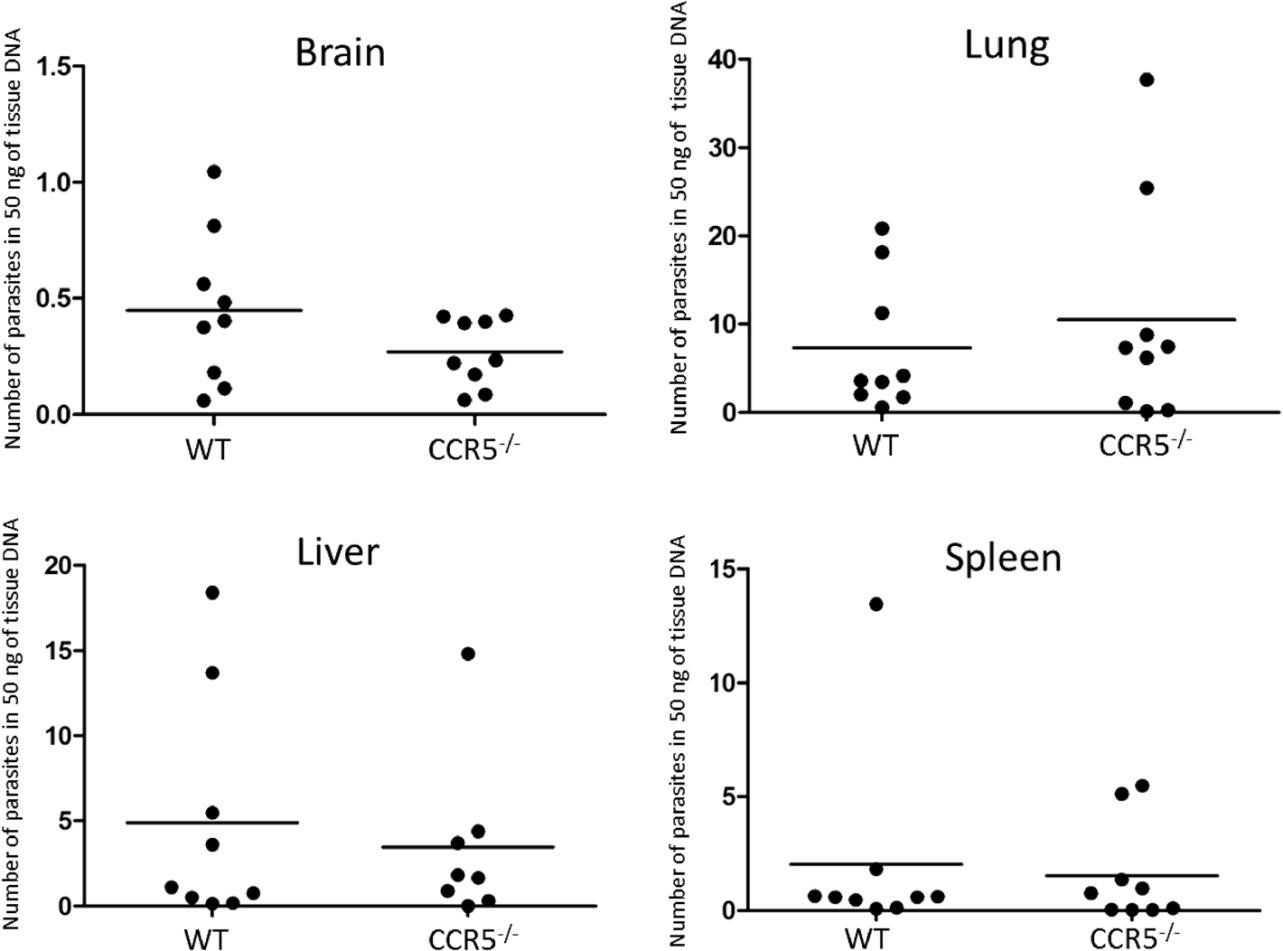
**Parasite burden in tissues.** The values are the number of parasites in 50 ng of tissue DNA. The number of parasites per individual (symbols) and mean levels (horizontal lines) are indicated (*N* = 9). Data were obtained from two independent experiments performed together. No significant difference was observed between the two groups by a student’s *t*-test.

Next, the number of parasites in brain, lung, liver and spleen tissues of mice at 5 day post-infection were measured by quantitative real-time PCR (Figure 2). As a result, no significant difference was found between tissue samples of the same organs from the two groups.

### Migration of peritoneal cells to the site of infection

**Figure 3:**
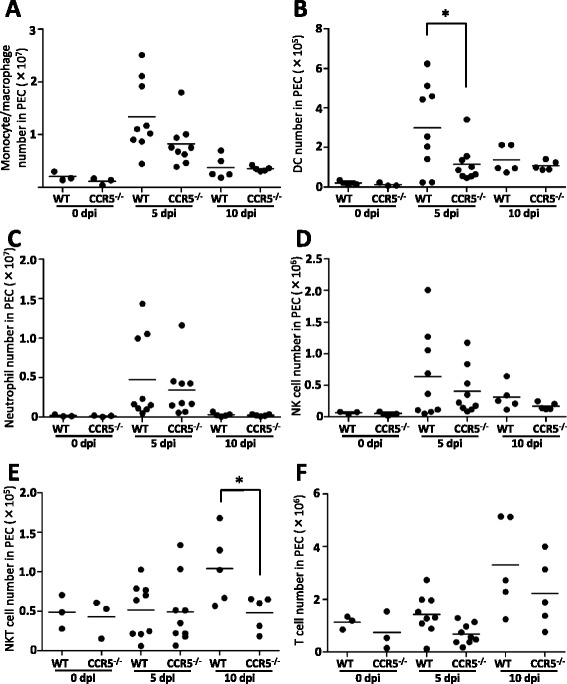
**Migration of peritoneal cells to the site of infection.** Peritoneal cells (PEC) were obtained from CCR5^−/−^ mice and C57BL/6 mice (wild-type, WT) at 0, 5 and 10 days post-infection (dpi) with 1 × 10^6^  *N. caninum* tachyzoites (0 dpi, *N* = 3; 5 dpi, *N* = 9 from two independent experiments; 10 dpi, *N* = 5). The cells were subjected to flow cytometry to determine the absolute number of monocytes/macrophages **(A)**, dendritic cells **(B)**, neutrophils **(C)**, NK cells **(D)**, NKT cells **(E)** and T cells **(F)**. Cell number per individual (symbols) and mean levels (horizontal lines) are indicated. Data were analyzed by a student’s *t*-test and compared with values taken on the same day post-infection. *P < 0.05.

The number of CD11b^+^ cells (monocytes and macrophages) that had migrated was not significantly different between the two groups (Figure 3A). In contrast, CD11c^+^ dendritic cells in the CCR5^−/−^ mice showed less migration than CD11c^+^ dendritic cells in the C57BL/6 mice at 5 days post-infection (Figure 3B). Similar migration dynamics for neutrophils, NK cells, and T cells were observed between the two groups, at 0, 5 and 10 days post-infection (Figures 3C, D, F). However, the NKT cells showed impaired migration in the CCR5^−/−^ mice at 10 days post-infection (Figure 3E). The flow cytometry results using *N. caninum* tachyzoites-expressing GFP showed that there was no significant difference in the infection rates and absolute numbers of the infected CD11b^+^ cells, CD11c^+^ cells, or CD3^+^ cells obtained from the peritoneal cells (Table 1).

**Table 1:** **Infection rates and number of CD11b** ^**+**^ **, CD11c** ^**+**^ **, or CD3** ^**+**^ **cells infected with** ***N. caninum*** **-expressing GFP**

		**Infection rates (%)**	**Infected cell number (×10** ^**4**^ **)**
CD11b^+^ cells	WT	2.03 ± 0.70, *P* = 0.255	9.68 ± 3.05, *P* = 0.73
	CCR5^−/−^	3.08 ± 1.93	9.09 ± 2.65
CD11c^+^ cells	WT	3.81 ± 1.01, *P* = 0.534	3.79 ± 1.27, *P* = 0.19
	CCR5^−/−^	4.23 ± 1.23	2.85 ± 1.07
CD3^+^ cells	WT	0.30 ± 0.18, *P* = 0.131	0.55 ± 0.22, *P* = 0.45
	CCR5^−/−^	0.80 ± 0.66	0.67 ± 0.31

Peritoneal cells were obtained from CCR5^−/−^ and C57BL/6 mice (WT) at 5 days after infection with 1 × 10^6^  *N. caninum* tachyzoites expressing GFP (*N* = 6). Cells were then subjected to flow cytometry to determine the infection rate and absolute number of monocytes/macrophages (CD11b^+^), dendritic cells (CD11c^+^) and T cells (CD3^+^) based on GFP^+^ cells. Data were analyzed by a student’s *t*-test. GFP: green fluorescent protein.

### Activation of macrophages and dendritic cells during *N. caninum* infection

**Figure 4:**
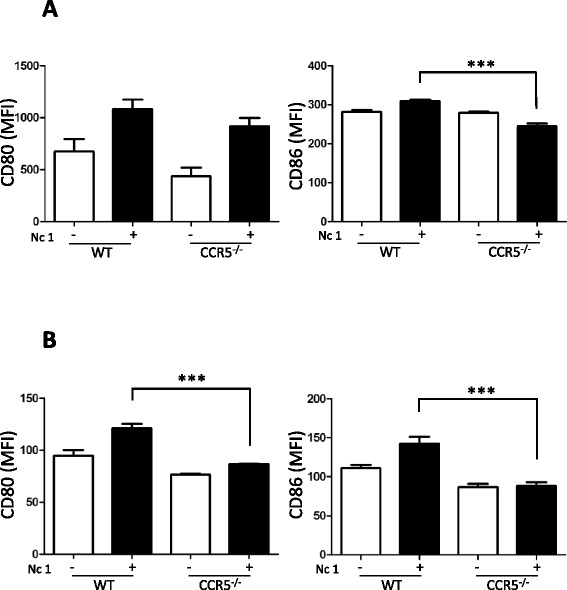
**Expression of cell surface markers on peritoneal macrophages and BMDCs.** The peritoneal macrophages **(A)** and BMDCs **(B)** were obtained from CCR5^−/−^ mice and C57BL/6 mice (wild-type, WT). Each value represents the mean fluorescence intensity (MFI) of the marker ± the standard deviation of three replicate samples. “–” indicates no stimuli and “+” indicates infection with *N. caninum* tachyzoites (Nc1). Data were analyzed by one-way ANOVA tests followed by Tukey’s multiple comparison. ***P < 0.001. Reproducibility of the data was confirmed by three independent experiments.

No difference in CD80 activation was observed, whereas there was significantly impaired CD86 activation in CCR5^−/−^ macrophages compared with the wild-type macrophages (Figure 4A). Additionally, in both cases, activation of CD80 and CD86 (costimulatory molecules) was significantly diminished in CCR5^−/−^ BMDCs upon *N. caninum* infection (Figure 4B). Although *N. caninum* infection triggered the production of IL-6 and IL-12p40 in the macrophages and BMDCs, there was no significant difference between wild-type and CCR5^−/−^ cells (data not shown).

### Measurement of inflammatory markers in liver and brain

**Figure 5:**
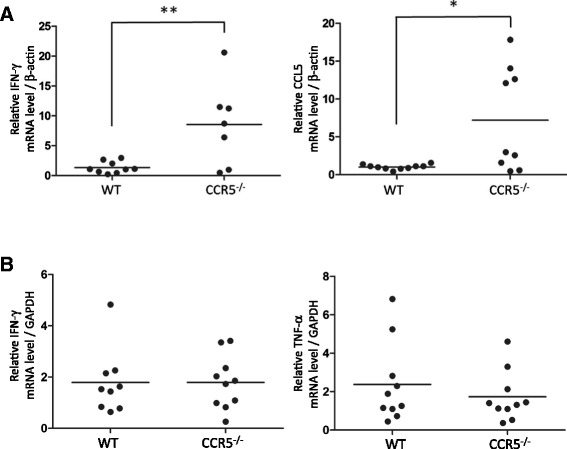
**Inflammatory marker expression in mouse brain and liver.** Brain and liver obtained from infected CCR5^−/−^ mice and C57BL/6 mice (wild-type, WT) at 5 days post-infection were prepared for measurement of their mRNA levels. The mRNA levels in brain **(A)** and liver **(B)** were standardized against the β-actin and GAPDH mRNA level values, respectively. The values per individual (symbols) and mean levels (horizontal lines) are indicated. Data were obtained from two independent experiments performed together. The individual with undetectable expression are not indicated. Data were analyzed by a student’s *t*-test. *P < 0.05.

At 5 days post-infection, IFN-γ, IL-6, IL-12p40 and nitric oxide (NO) levels were not significantly different in the serum and the ascites fluid between the wild-type and CCR5^−/−^ mice (data not shown). At 5 days post-infection, IFN-γ and CCL5 mRNA levels in the brains of the infected CCR5^−/−^ mice were significantly higher than those of the infected wild-type animals (Figure 5A); however, there was no significant difference in the IFN-γ and TNF-α expression levels in the liver (Figure 5B).

### Pathological analysis of infected mice

**Figure 6:**
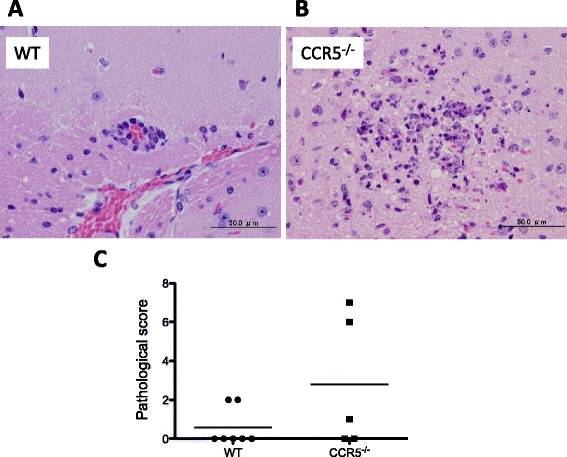
**Pathological analysis of brain tissue.** Slight to mild lesions including perivascular cuffs were observed in the C57BL/6 mice **(A)**, and slight to moderate lesions including necrotic focus with glial cell infiltration were observed in the CCR5^−/−^ mice **(B)**. Additionally, the severity of the histopathological lesions was analyzed **(C)**. No significant difference was observed in the pathological score between the two groups (student’s *t*-test).

We performed a pathological analysis of liver, spleen, kidney, heart, lung and brain at 5 days post-infection. In liver, mononuclear cell infiltration was observed in both groups of mice (five mice per group) while some CCR5^−/−^ mice showed focal necrosis (data not shown). However, there were no significant findings in the other organs. Next, pathological change of brain at 8 days post-infection was examined. Two of nine C57BL/6 mice and four of nine CCR5^−/−^ mice died before their brains were collected. While only slight to mild lesions, including perivascular cuffs, were observed in the brains of two C57BL/6 mice (Figure 6A), slight to moderate lesions including glial cell infiltration and meningitis were observed in the brains of the CCR5^−/−^ mice (Figure 6B). Although some mice in the CCR5^−/−^ group had a high pathological score (Figure 6C) and a high parasite load in their brain data not shown, no statistically significant difference was observed between the CCR5^−/−^ and C57BL/6 groups.

### Microglia activation during *N. caninum* infection

**Figure 7:**
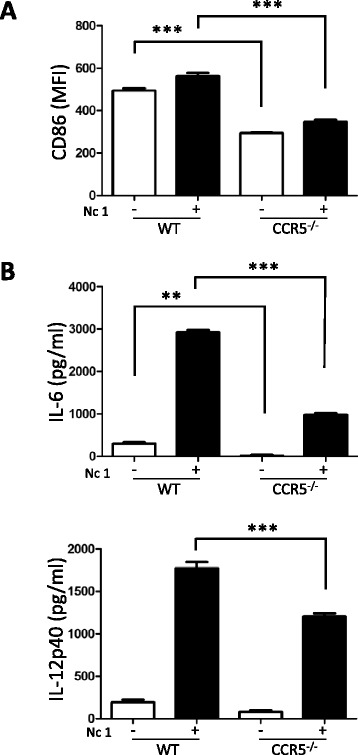
**Expression of cell surface markers and cytokine production in microglia. (A)** Each value represents the mean fluorescence intensity (MFI) of the marker ± the standard deviation of four replicate samples. “–” indicates no stimuli and “+” indicates infection with *N. caninum* tachyzoites (Nc1). **(B)** IL-6 and IL-12p40 in the culture supernatant were analyzed by cytokine ELISA. Each value represents the mean ± standard deviation of four replicate samples. Data were analyzed by one-way ANOVA tests followed by Tukey’s multiple comparison. **P < 0.01, ***P < 0.001. Reproducibility of the data was confirmed by two independent experiments.

As shown in Figure 7A, CD86 expression was significantly lower in CCR5^−/−^ microglia with or without infection while no significant difference in activation of MHC class II was observed (data not shown). Moreover, IL-6 and IL-12p40 production levels in CCR5^−/−^ microglia were significantly lower than those in wild-type cells during *N. caninum* infection (Figure 7B). In contrast, there was no significant difference in IL-6 production between wild-type and CCR5^−/−^ primary astrocytes (data not shown).

## Discussion

In the present study, we showed that CCR5^−/−^ mice experienced increased mortality during *N. caninum* infection compared with C57BL/6 mice. It has been shown that CCR5 is crucially involved in the pathway underlying resistance to *T. gondii* because of its ability to induce IL-12 production by dendritic cells [10,18]. Additionally, CCR5^−/−^ mice have been shown to display enhanced parasite burden and mortality during *T. gondii* infection [19]. These results suggest that CCR5 plays a physiological role in immunology and inflammation during parasite infection. Alternatively, antigen-presenting cells may transport intracellular pathogens such as *N. caninum* and *T. gondii* away from the sites of primary infection and help them to propagate inside the host [12,20]. However, in the present study, no difference in the parasite load in the organs of CCR5^−/−^ and wild-type mice was seen at 5 and 8 days post-infection with *N. caninum*. This result suggests that parasite burden does not contribute to death in CCR5^−/−^ mice during *N. caninum* infection.

It is well-known that rapid recruitment of monocytes/macrophages to the sites of infection has potential to enhance the innate immune responses of the host against pathogens. Our recent results showed that macrophage-depleted mice exhibited increased sensitivity to *N. caninum* infection [21]. However, migration of monocytes/macrophages to the site of infection was not significantly different between CCR5^−/−^ and wild-type mice. Additionally, CCR5^−/−^ macrophage activation upon infection with *N. caninum* was similar to that of the wild-type cells, with the exception of CD86 expression levels. Therefore, other factors may play a role in the increased mortality of CCR5^−/−^ mice during infection with *N. caninum*.

Significant differences were observed in the migration of dendritic cells and NKT cells at 5 and 10 days post-infection, respectively, indicating that these cells migrated to the sites of infection in a CCR5-dependent manner. Our group previously showed that depletion of NKT cells, but not NK cells, increased the parasite burden in mouse brains and inhibited the activation of CD4^+^ cells, suggesting that NKT cells play a crucial role in protection against the early stage of *N. caninum* infection [22]. Thus, NKT cells may contribute to CCR5-dependent protective immunity. The main role of dendritic cells is antigen presentation; therefore, impairment of dendritic cell migration and activation suppresses the induction of antigen presentation in the lymph nodes, leading to down-regulation of adaptive immunity [6]. CD80 and CD86 expression levels in the CCR5^−/−^ dendritic cells were significantly lower than those in the wild-type cells. This suggests that CCR5-mediated activation of dendritic cells and NKT cells in response to *N. caninum* may be partially involved in protective immunity despite the similar parasite burden and infection rates in the tissue or cells between the CCR5^−/−^ and wild-type mice.

Interestingly, brain (but not liver) from the *N. caninum*-infected CCR5^−/−^ mice showed more severe tissue damage and increased inflammation than the same tissue from the infected wild-type animals, despite no significant difference in the parasite load between the groups. Glial cells such as astrocytes and microglia play an important role in brain homeostasis. Astrocytes support neuronal function by secreting neuropoietic factors such as IL-6 [23]. However, no significant production of IL-6 between wild-type and CCR5^−/−^ primary astrocytes was seen upon infection with *N. caninum*. Microglia cells are responsible for initial immune defenses in the central nervous system (CNS). In response to tissue injury or pathogen infection, microglia proliferate and secrete pro- and anti-inflammatory cytokines, prostaglandins and free radicals [24]. Microglia appear to be the major effector cells that inhibit *T. gondii* tachyzoite proliferation in the brain via TNF-α, IL-6 or NO [25,26]. Microglia, which act like macrophages or dendritic cells in the brain, produce the cytokines necessary for recruitment and activation of T cells to control *T. gondii* infection [27]. In the present study, in comparison with the wild type microglia, CCR5^−/−^ cells showed lower expression levels of CD86 and impaired production of IL-6 and IL-12 p40 against *in vitro* infection with *N. caninum*, suggesting that CCR5^−/−^ microglia were unable to trigger neuroprotection or the level of protective immunity required to clear parasites from the brain. Moreover, CCR5 and its ligands are expressed in microglia and neurons, respectively, in response to nerve injury, suggesting that CCR5-mediated neuron-glia signaling protects neurons by suppressing microglia toxicity [28]. Although the ability of IFN-γ to reduce parasite numbers is well-known [27], overproduction of it causes tissue damage [19]. CCL5, one of the ligands for CCR5, is expressed in response to inflammation following T cell recruitment [29]. In the present study, IFN-γ and CCL5 mRNA expression in brain tissue was significantly elevated in the CCR5^−/−^ mice compared with that of the wild-type mice; however, no significant difference was observed in IFN-γ and TNF-α mRNA levels in liver tissue between these groups of mice, indicating that there was more severe damage to the brains of infected CCR5^−/−^ mice. Thus, brain damage caused by microglia dysfunction might result in the earlier onset of neurological signs in CCR5^−/−^ mice after infection with *N. caninum*.

Neurological signs are a typical feature of neosporosis. In most CNS diseases, CCR5 deletion is deleterious to the host; infectious agents for which this has been shown include *Cryptococcus neoformans* [30], herpes simplex virus [31,32], and West Nile Virus [33]. In contrast, CCR5-deficiency in mice diminished susceptibility to infection with *Plasmodium berghei* (ANKA strain) by reducing CD8^+^ T cell accumulation and T-helper 1 cytokine production in the brain [9]. If, in some cases, CCR5 represents a susceptibility factor for the spread of pathogens in the brain, in others it confers resistance against the development of severe disease. To better understand the physiological role of CCR5, the function of its ligand should be considered. Interestingly, *T. gondii* possesses a unique molecule for stimulating immune responses and cell migration in the host. TgCyp18 appears to induce IL-12 production by interacting directly with CCR5 [11,18,34]. Moreover, overproduction of TgCyp18 regulates host cell migration and enhances parasite dissemination in a CCR5-independent manner [35]. *N. caninum* also has a cyclophilin gene. However, *N. caninum*-derived cyclophilin (NcCyp) appears to contribute to host cell migration in a CCR5-dependent way [13]. Therefore, the complex reactions underlying the development of neosporosis and the involvement of CCR5 and NcCyp in immune and nervous system reactions should be investigated further. It is likely that such studies will make important contributions to the understanding of host-parasite interactions.

Although ruminants are clinically affected by *N. caninum* infection, cattle generally show few clinical symptoms following the infection. Specific antibody and cell-mediated immune responses have been observed in both naturally infected cattle and those experimentally infected with *N. caninum*. It is important also to consider the differences in immune responses between pregnant and non-pregnant cattle, since pregnancy can modulate the immune responses against *N. caninum* [36]. In early pregnancy, strong Th1 immune responses against the parasite antigen at the maternal-foetal interface may induce abortion. Thus, a Th1 immune response is thought to be detrimental to pregnancy [37,38]. Th1 immune responses at the maternal-foetal interface including CD4^+^ cell infiltration and IFN–γ expression have been associated with tissue destruction in early or mid- gestation [39,40]. Therefore, migration of inflammatory cells at the maternal-foetal interface may trigger the *N. caninum*-induced abortion. Our previous study showed that recombinant NcCyp caused the CCR5-dependent migration of bovine peripheral blood mononuclear cells [13]. This result suggests that CCR5-dependent immunity may be involved in bovine abortion following *N. caninum* infection.

## Conclusions

Our findings indicate that migration and activation of immune cells via CCR5 is required for controlling *N. caninum* parasites during the early phase of the infection. Our data suggest that dendritic cells and microglia play a role in CCR5-mediated protectve immunity against *N. caninum*. Hence, it is important to consider the contribution that the parasite-derived molecule such as NcCyp plays in CCR5-dependent host immunity.

## Additional file

Additional file 1: Table S1. List of monoclonal antibody used in this study.

## Abbreviations

CCR5: C-C chemokine receptor 5
CCL5: C-C ligand 5
NK cell: Natural killer cell
NKT cell: Natural killer T cell
MIP-1α: Macrophage inflammatory protein-1 alpha
MIP-1β: Macrophage inflammatory protein-1 beta
TgCyp18: *Toxoplasma gondii* cyclophilin 18
EMEM: Eagle’s minimum essential medium
FBS: Fetal bovine serum
UNG: Urasil-N-glycosilase
Ct: Cycle threshold
GAPDH: Glyceraldehyde-3-phosphate dehydrogenase
mAb: Monoclonal antibody
PE: Phycoerythrin
BMDC: Bone marrow-derived dendritic cell
GM-CSF: Granulocyte-macrophage colony-stimulating factor
NO: Nitric oxide
CNS: Central nervous system
NcCyp: *Neospora caninum*-derived cyclophilin
WT: Wild-type
PEC: Peritoneal cell
dpi: Days post-infection
MFI: Mean fluorescence intensity
