# Supplementary material for: Role of the chemokine receptor CCR5-dependent host defense system in Neospora caninum infections
Source: Parasit Vectors. 2015 Jan 6;8:5. doi: 10.1186/s13071-014-0620-5 (PMC4455913; doi:10.1186/s13071-014-0620-5)
Supplement: Additional file 1: Table S1. — List of monoclonal antibody used in this study. [file 13071_2014_620_MOESM1_ESM.docx]

Additional file 1: Table S1. List of monoclonal antibody used in this study

| Antibody name | Clone | Company |
| --- | --- | --- |
| FITC-conjugated anti-mouse CD11b | M1/70 | BD Pharmingen (San Diego, CA, USA) |
| FITC-conjugated anti-mouse CD11c | HL3 | BD Pharmingen |
| FITC-conjugated anti-mouse CD3e | 145-2C11 | BD Pharmingen |
| FITC-conjugated anti-mouse Ly-6G (Gr-1)  FITC-conjugated anti-mouse F4/80 | RB6-8C5  BM8 | eBioscience (San Diego, CA, USA)  eBioscience |
| phycoerythrin (PE)-conjugated anti-mouse CD11b | M1/70 | BD Pharmingen |
| PE-conjugated anti-mouse CD11c | HL3 | BD Pharmingen |
| PE-conjugated anti-mouse NK1.1  PE-conjugated anti-mouse I-A^b^  PE-conjugated anti-mouse CD80  PE-conjugated anti-mouse CD86  anti-mouse CD16/CD32 (FcBlock™) | PK136  AF6-120.1  16-10A1  GL1  2.4G2 | BD Pharmingen  BD Pharmingen  BD Pharmingen  BD Pharmingen  BD Pharmingen |
